# Editorial: Secondary hyperparathyroidism: an ongoing challenge for the nephrologist

**DOI:** 10.3389/fmed.2023.1305791

**Published:** 2023-11-23

**Authors:** Claudia Torino, Rocco Tripepi, Domenico Russo, Antonio Demetrio Vilasi, Vincenzo Antonio Panuccio

**Affiliations:** ^1^Institute of Clinical Physiology, National Research Council, Reggio Calabria, Italy; ^2^Clinical, Medical, Surgical Department of Nephrology, University of Naples, Naples, Italy; ^3^GOM “Bianchi-Melacrino-Morelli, Reggio Calabria, Italy

**Keywords:** hyperparathyroidism, chronic kidney disease, ESRD, nephrology, treatment

Hyperparathyroidism is a clinical condition caused by an excessive secretion of parathormone (PTH) by parathyroid glands. It can be classified as primary, secondary, and tertiary. In primary hyperparathyroidism, the hyper-function of parathyroid glands is due to hyperplasia, carcinoma, or adenoma and can be associated with multiple endocrine neoplasia. In this case, serum calcium levels are high and are associated with normal or low serum phosphate levels. Primary hyperparathyroidism is often asymptomatic; thus, the disease is commonly identified once incidental, asymptomatic hypercalcemia (often associated with nephrolithiasis) is detected. Secondary hyperparathyroidism is due to a physiological stimulation of parathyroid glands in response to hypocalcaemia, usually consequent to chronic kidney disease (CKD) or other possible causes of hypovitaminosis D. Serum calcium levels are generally normal or low and are associated with hyperphosphatemia. Hyperparathyroidism is defined as “tertiary” when it is longstanding or persists even after successful renal transplantation. It is associated with high serum calcium and phosphate levels.

Secondary hyperparathyroidism (SHPT) is a pervasive complication in CKD patients, with a prevalence increasing in parallel with the worsening of kidney function (1). Its genesis is multifactorial, with dysregulation of Vitamin D and FGF-23 playing a major role (2). In patients affected by hyperparathyroidism, the skeleton loses its natural strength, becoming extremely frail and subject to fractures (3, 4) (Figure 1). This condition, together with the other complications of hyperparathyroidism, contribute to reduce the quality of life in these patients and also increases the risk of cardiovascular morbidity and mortality (5, 6).

**Figure 1:**
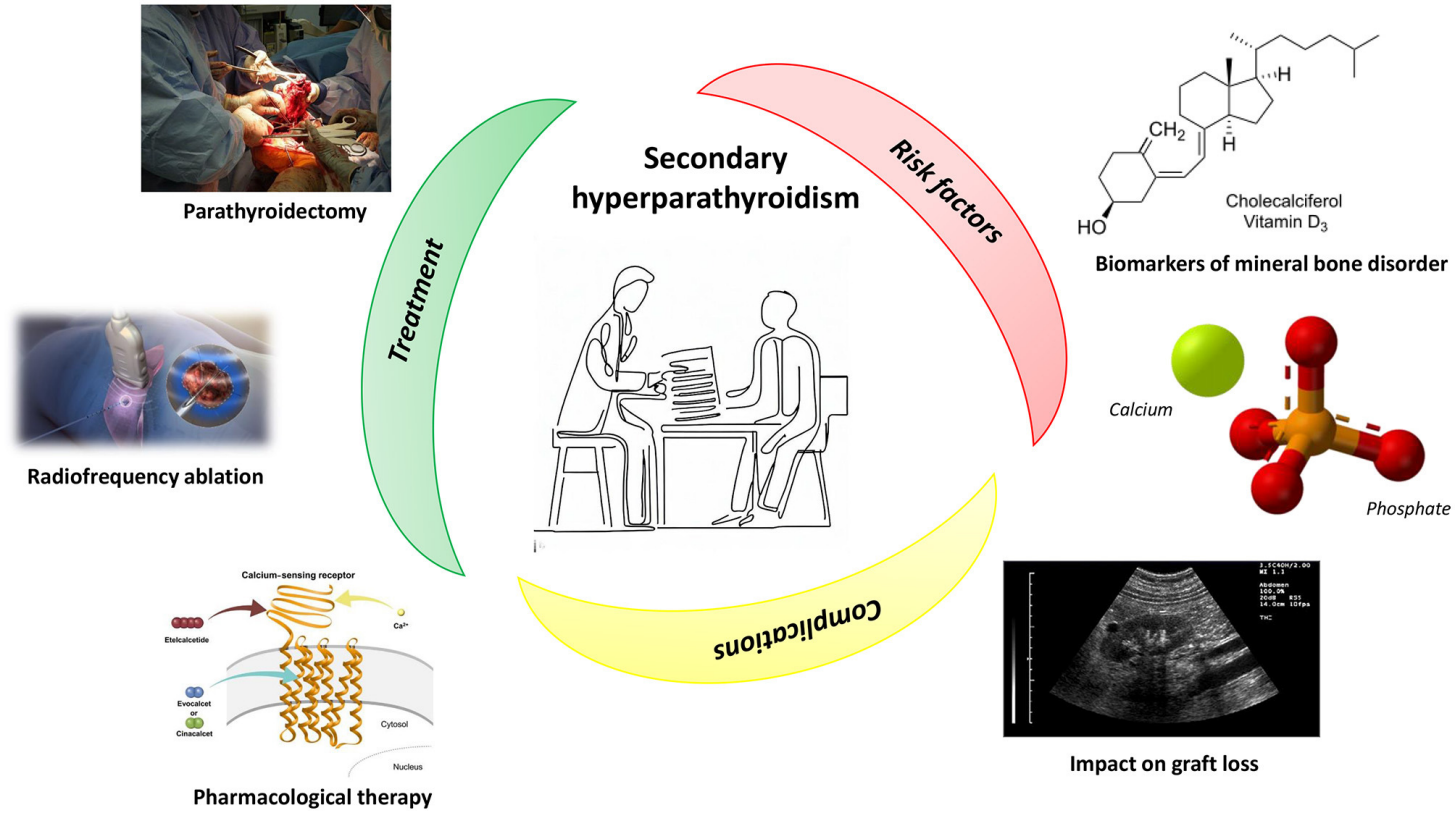
Secondary hyperparathyroidism: treatment, risk factors, and complications. See the text for more details. Image sources are: Image at the center of the figure: Reprinted with permission from RMHare, licensed under CC0 1.0, https://commons.wikimedia.org/wiki/File:Doctor_showing_form.jpg. Parathyroidectomy: Reprinted with permission from BennyK95, work in the Public Domain, https://commons.wikimedia.org/wiki/File:RightFemurII.JPG. Radiofrequency ablation: Reprinted with permission from Scientific Animations (https://www.scientificanimations.com/wiki-images/), licensed under CC BY-SA 4.0, https://www.scientificanimations.com/wp-content/uploads/2018/06/Radiofrequency-ablation.jpg. Pharmacological therapy: Reprinted with permission from Figure 2 of “Evocalcet: A New Oral Calcimimetic for Dialysis Patients With Secondary Hyperparathyroidism” by Akizawa et al., licensed under CC BY-NC 4.0, https://doi.org/10.1111/1744-9987.13434. Biomarkers of mineral bone disorder: Reprinted with permission from Hbf878 (https://commons.wikimedia.org/wiki/User:Hbf878), licensed under CC0 1.0, https://commons.wikimedia.org/wiki/File:Vitamin_D_biosynthesis_in_fungi_and_animals.svg. Calcium and Phosphate image: Reprinted with permission from Hoa112008, work in the Public Domain, https://commons.wikimedia.org/wiki/File:Tricalcium-phosphate-3D-balls-ionic.png. Impact on graft loss: Reprinted with permission from Schomynv (https://commons.wikimedia.org/wiki/User:Schomynv), work in the Public Domain, https://commons.wikimedia.org/wiki/File:Kidney_with_THI.jpg.

Total parathyroidectomy (PTx) (Figure 1) represents one of the therapeutic options for the treatment of secondary hyperparathyroidism. In the paper by Disthabanchong et al. it was retrospectively examined whether this approach was able to improve nutritional status in a cohort of haemodialysis (HD) patients. The authors found that nutritional impairment was pervasive in patients with severe hyperparathyroidism, but such a condition improved after PTx, as witnessed by nutritional indices, which became comparable to those of patients with no or mild hyperparathyroidism.

In order for PTx to be effective, the removal of all the parathyroid glands is needed. It is not uncommon after surgery to have persistent high PTH levels, meaning that there is one or more glands left. These remaining parts may be stimulated, leading to an excessive secretion of PTH (7, 8). In the paper by Hiramitsu et al. preoperative and intraoperative factors contributing to successful PTx were investigated. The analysis showed that intraoperative intact PTH (IOIPTH) monitoring and the number of parathyroid glands (PTGs) identified in frozen section diagnosis were the only factors contributing to successful PTx. More specifically, IOIPTH monitoring was useful in patients with <4 PTGs identified during PTx.

Hypocalcaemia is common after parathyroidectomy, with a duration depending on the extent of the bone disease. In CKD patients, it can be severe and prolonged, despite parathyroid hormone levels. This condition is known as “hungry bone syndrome,” and it is common especially in patients with concomitant osteitis fibrosa. It associates with other electrolytes alterations, such as low serum phosphate and magnesium and high serum potassium levels. The management of hypocalcaemia after PTx is crucial to avoid potential complications, such as tetanic episodes or arrhythmia. The first therapeutic step to obtain serum calcium stability consists in intravenous calcium supplements; this can be switched into oral therapy associated with vitamin D supplements. In this phase, it is fundamental that patients are closely monitored.

Although PTx represents the election choice for the treatment of severe SHPT (9), this approach is not always applicable in SHPT patients with severe comorbidities because of the high surgical risk. An alternative to surgery has been proposed by Muhetaer et al. who report the case of a young man affected by secondary hyperparathyroidism and who underwent radiofrequency ablation to eliminate seven parathyroid glands hyperplasia (Figure 1). After the treatment, the patient's bone pain disappeared, the level of PTH was stably between 136 and 242 pg/ml, and the quality of his life improved significantly. Based on this patient's experience, the authors conclude that, in case of patients with cardiopulmonary dysfunction, who cannot perform surgery, radiofrequency ablation can be widely used, even though a longer follow-up and clinical indicators are needed to comprehensively determine the clinical efficacy.

Similarly, Panuccio et al. described the case of another young man on chronic haemodialysis and SHPT who developed pancytopenia with resistant anemia. Once treated with etelcalcetide, an improvement in SHPT concomitant with near normalization of blood counts was observed. This is the first case in which etelcalcetide treatment seems comparable to parathyroidectomy on SHPT and is associated with significant improvement in severe myelofibrosis, paving the way for future studies in this field.

Vitamin D is one of the players involved in the development of SHPT. However, if its role in SHPT progression has been unraveled, less known is the role of vitamin D deficiency in the onset of SHPT and mineral bone disorder in CKD patients. To this purpose, Daimon et al. studied 930 participants in a population-based Iwaki study in Japan. The results of this analysis showed a significant correlation between estimated glomerular filtration rate (eGFR) and serum intact PTH (iPTH) concentration in patients with vitamin D deficiency, suggesting a possible role of vitamin D deficiency in PTH increase in CKD patients and could be useful in the future for targeting SHPT therapy.

Vitamin D has also shown to be a predictor of renal worsening function in CKD patients, as illustrated by Galassi et al.. In the frame of the PASCaL-1,25D study, an observational, prospective, monocentric study, the authors investigated the capability of 1,25(OH)_2_D to predict PTH increase and worsening of kidney function in CKD patients. The preliminary analysis, performed on 71 patients, showed a reverse, significant association between PTH and 1,25(OH)_2_D levels. Higher 1,25(OH)_2_D levels were associated with a reduced risk of worsening of kidney function at univariate analysis. In addition, low 1,25(OH)_2_D was highly sensitive in predicting worsening of kidney function in CKD stage 3 and in non-elderly patients. These results highlight the potential usefulness of 1,25(OH)_2_D as a predictor of the worsening of kidney function in non-elderly patients with stage-3 CKD. However, further studies are needed to confirm this preliminary research.

If the impact of hyperparathyroidism on morbidity and mortality in CKD patients has been established, its role in graft loss after kidney transplant is still a matter of debate (Figure 1). To unravel this issue, Molinari et al. retrospectively evaluated the association between PTH levels and long-term graft loss in 871 renal transplant recipients. They found a high prevalence of SHPT after transplantation, with pre-RTx Cinacalcet therapy, uric acid levels, and hypovitaminosis-D as risk factors for the disease. Interestingly, high PTH levels during the first year of transplantation were associated with long-term graft loss, confirming findings already reported in the literature.

It is known that, in CKD patients, biomarkers of mineral bone disorder are associated with endothelial dysfunction, but it is not known if they are able to induce endothelial dysfunction also with normal phosphorous levels. Lee and Kim unraveled this issue in a cross-sectional study, performed in CKD patients with normal phosphorus levels, in whom endothelial function was measured. The authors found an inverse relationship between eGFR and both phosphorus levels and PTH. Furthermore, both iPTH and phosphorus levels were associated with endothelial dysfunction, and this association held true even after adjustment for covariates, including eGFR. Mediation analysis showed a mediation effect by iPTH on the associations between eGFR, phosphorus, and endothelial dysfunction. The authors concluded that serum phosphorous, even in a normal range, and elevated PTH values are associated with endothelial dysfunction in non-dialysis CKD patients.

During recent years, pharmaceutical companies have developed new drugs that can pave the way to personalized medicine for SHPT patients. In particular, calcimimetics have been demonstrated to be effective in controlling hyperparathyroidism; studies are also underway to evaluate the effect on extra-skeletal complications, such as cardiovascular comorbidities. Another very promising category of drugs are antisclerostin antibodies; a short formal indication is awaited for their use also in CKD patients. Recent progress gives rise to hope that secondary hyperparathyroidism may 1 day be appropriately treated. However, further studies are still required to explore the new pharmacological options for the treatment of SHPT.

## Author contributions

CT: Conceptualization, Writing—original draft, Writing—review & editing. RT: Writing—review & editing. DR: Writing—review & editing. AV: Writing—review & editing. VP: Conceptualization, Writing—review & editing.

## References

[1] Bozic M Diaz-Tocados JM Bermudez-Lopez M Forné C Martinez C Fernandez E. Independent effects of secondary hyperparathyroidism and hyperphosphataemia on chronic kidney disease progression and cardiovascular events: an analysis from the NEFRONA cohort. Nephrol Dial Transp. (2022) 37:184. 10.1093/ndt/gfab184 34021359

[2] Jean G Souberbielle JC Chazot C. Vitamin D in chronic kidney disease and dialysis patients. Nutrients. (2017) 9:328. 10.3390/nu9040328 28346348 PMC5409667

[3] Jadoul M Albert JM Akiba T Akizawa T Arab L Bragg-Gresham JL. Incidence and risk factors for hip or other bone fractures among hemodialysis patients in the dialysis outcomes and practice patterns study. Kidney Int. (2006) 70:1358–66. 10.1038/sj.ki.5001754 16929251

[4] Torres A Lorenzo V Hernández D Rodríguez JC Concepción MT Rodríguez AP. Bone disease in predialysis, hemodialysis, and CAPD patients: evidence of a better bone response to PTH. Kidney Int. (1995) 47:1434–42. 7637272 10.1038/ki.1995.201

[5] Block GA Klassen PS Lazarus JM Ofsthun N Lowrie EG Chertow GM. Mineral metabolism, mortality, and morbidity in maintenance hemodialysis. J Am Soc Nephrol. (2004) 15:1–2. 10.1097/01.ASN.0000133041.27682.A2 15284307

[6] Tentori F Blayney MJ Albert JM Gillespie BW Kerr PG Bommer J. Mortality risk for dialysis patients with different levels of serum calcium, phosphorus, and PTH: the dialysis outcomes and practice patterns study (DOPPS). Am J Kid Dis Off J Nat Kid Found. (2008) 52:519–30. 10.1053/j.ajkd.2008.03.020 18514987

[7] Hibi Y Tominaga Y Sato T Katayama A Haba T Uchida K. Reoperation for renal hyperparathyroidism. World J Surg. (2002) 26:1301–7. 10.1007/s00268-002-6731-8 12205559

[8] Tominaga Y Katayama A Sato T Matsuoka S Goto N Haba T. Re-operation is frequently required when parathyroid glands remain after initial parathyroidectomy for advanced secondary hyperparathyroidism in uraemic patients. Nephrol Dial Transp Off Pub Eur Dial Trans Assoc Eur Renal Assoc. (2003) 18:65–70. 10.1093/ndt/gfg1017 12771305

[9] Ketteler M Block GA Evenepoel P Fukagawa M Herzog CA McCann L. Executive summary of the 2017 KDIGO chronic kidney disease-mineral and bone disorder (CKD-MBD) guideline update: what's changed and why it matters. Kidney Int. (2017) 92:26–36. 10.1016/j.kint.2017.04.006 28646995

